# Dual Actions of Mammalian and Piscine Gonadotropin-Inhibitory Hormones, RFamide-Related Peptides and LPXRFamide Peptides, in the Hypothalamic–Pituitary–Gonadal Axis

**DOI:** 10.3389/fendo.2017.00377

**Published:** 2018-01-11

**Authors:** Takayoshi Ubuka, Ishwar Parhar

**Affiliations:** ^1^Jeffrey Cheah School of Medicine and Health Sciences, Brain Research Institute Monash Sunway, Monash University Malaysia, Sunway, Malaysia

**Keywords:** gonadotropin-releasing hormone, GPR147, aromatase, neuroestrogen, GPR30, receptor heteromerization, receptor internalization, sex steroids

## Abstract

Gonadotropin-inhibitory hormone (GnIH) is a hypothalamic neuropeptide that decreases gonadotropin synthesis and release by directly acting on the gonadotrope or by decreasing the activity of gonadotropin-releasing hormone (GnRH) neurons. GnIH is also called RFamide-related peptide in mammals or LPXRFamide peptide in fishes due to its characteristic C-terminal structure. The primary receptor for GnIH is GPR147 that inhibits cAMP production in target cells. Although most of the studies in mammals, birds, and fish have shown the inhibitory action of GnIH in the hypothalamic–pituitary–gonadal (HPG) axis, several *in vivo* studies in mammals and many *in vivo* and *in vitro* studies in fish have shown its stimulatory action. In mouse, although the firing rate of the majority of GnRH neurons is decreased, a small population of GnRH neurons is stimulated by GnIH. In hamsters, GnIH inhibits luteinizing hormone (LH) release in the breeding season when their endogenous LH level is high but stimulates LH release in non-breeding season when their LH level is basal. Besides different effects of GnIH on the HPG axis depending on the reproductive stages in fish, higher concentration or longer duration of GnIH administration can stimulate their HPG axis. These results suggest that GnIH action in the HPG axis is modulated by sex-steroid concentration, the action of neuroestrogen synthesized by the activity of aromatase stimulated by GnIH, estrogen membrane receptor, heteromerization and internalization of GnIH, GnRH, and estrogen membrane receptors. The inhibitory and stimulatory action of GnIH in the HPG axis may have a physiological role to maintain reproductive homeostasis according to developmental and reproductive stages.

## Introduction

Gonadotropin-inhibitory hormone (GnIH) is a hypothalamic neuropeptide that was initially isolated from the brain of Japanese quail, which decreases luteinizing hormone (LH) concentration in the culture medium of the anterior pituitary gland (1). *In vivo* administration of quail GnIH also decreases gonadotropin synthesis as well as gonadal development and maintenance in quail (2). The C-terminal of GnIH peptides has an LPXRFamide (LPXRFa, X = L or Q) motif. Therefore, peptides orthologous to GnIH are also called RFamide-related peptide (RFRP) in mammals and LPXRFa peptides in non-mammalian and non-avian vertebrates (3). Most of the studies in mammals, birds, and fish have shown inhibitory effects of GnIH on the hypothalamic–pituitary–gonadal (HPG) axis; however, several *in vivo* and *in vitro* studies in mammals and fish show its stimulatory effects (3, 4). Here, we highlight studies that show stimulatory effects of GnIH on the HPG axis and investigate their physiological or pharmacological mechanisms.

## Endogenous Mature GnIH Peptides

Human RFRP-1 and -3 (5), macaque RFRP-3 (6), Siberian hamster RFRP-1 and -3 (7), rat RFRP-3 (8), bovine RFRP-1 (9) and -3 (10), European starling GnIH (11), zebra finch GnIH (12), chicken GnIH (13), quail GnIH (1), quail GnIH-related peptide (RP) 2 (14), red-eared slider LPXRFamide-1, 2, 3 (15), frog growth hormone-releasing hormone (fGRP), fGRP-RP-1, fGRP-RP-2, and fGRP-RP-3 (16, 17), Japanese red-bellied newt LPXRFa-1, -2, -3, -4 (18), and goldfish LPXRFa-3 (19) are identified as endogenous mature LPXRFa peptides by cDNA sequencing, immunoaffinity chromatography, and mass spectrometry in gnathostomes (3). Lamprey is a jawless fish that is one of the most primitive among vertebrates. Lamprey LPXRFamide peptide precursor gene encompasses C-terminal QPQRFamide (LPXRFa-1a, 1b) and RPQRFamide peptides (LPXRFa-2) that have been identified by mass spectrometry (20). LPXRFamide peptide precursor gene is also found in amphioxus, one of the most primitive chordates (protochordates), which encompasses three mature C-terminal RPQRFamide peptides (PQRFa-1, PQRFa-2, and PQRFa-3) (21). Identified and putative amino-acid sequences of GnIH peptides are summarized in Table 1. Although the C-terminal LPXRFa structure is key for binding of GnIH to its receptor (22), the N-terminal structure may modify the action of GnIH. Studies are needed to investigate the function of the N-terminal of GnIH and the differential effect of orthologous LPXRFa peptides encoded in the precursor polypeptide (Table 1).

**Table 1:** Amino-acid sequences of RFRPs, GnIHs, and LPXRFa peptides in chordates.

	Animal	Name	Sequence	Reference
Mammals	Human	RFRP-1	MPHSFANLPLRFa	(5)
		RFRP-3	VPNLPQRFa	(5)
	Macaque	RFRP-1^a^	MPHSVTNLPLRFa	(6)
		RFRP-3	SGRNMEVSLVRQVLNLPQRFa	(6)
	Bovine	RFRP-1	SLTFEEVKDWAPKIKMNKPVVNKMPPSAANLPLRFa	(9)
		RFRP-3	AMAHLPLRLGKNREDSLSRWVPNLPQRFa	(10)
	Horse	RFRP-3^a^	IPNLPQRFa	(23)
	Rat	RFRP-1^a^	SVTFQELKDWGAKKDIKMSPAPANKVPHSAANLPLRFa	(8)
		RFRP-3	ANMEAGTMSHFPSLPQRFa	(8)
	Siberian hamster	RFRP-1	SPAPANKVPHSAANLPLRFa	(7)
		RFRP-3	TLSRVPSLPQRFa	(7)
	Syrian hamster	RFRP-1^a^	VPHSAANLPLRFa	(45)
		RFRP-3^a^	VPSLPQRFa	(45)

Birds	Quail	GnIH	SIKPSAYLPLRFa	(1)
		GnIH-RP-1^a^	SLNFEEMKDWGSKNFMKVNTPTVNKVPNSVANLPLRFa	(14)
		GnIH-RP-2	SSIQSLLNLPQRFa	(14)
	Chicken	GnIH	SIRPSAYLPLRFa	(13)
		GnIH-RP-1^a^	SLNFEEMKDWGSKNFLKVNTPTVNKVPNSVANLPLRFa	(24)
		GnIH-RP-2^a^	SSIQSLLNLPQRFa	(24)
	White-crowned sparrow	GnIH^a^	SIKPFSNLPLRFa	(62)
		GnIH-RP-1^a^	SLNFEEMEDWGSKDIIKMNPFTASKMPNSVANLPLRFa	(62)
		GnIH-RP-2^a^	SPLVKGSSQSLLNLPQRFa	(62)
	European starling	GnIH	SIKPFANLPLRFa	(11)
		GnIH-RP-1^a^	SLNFDEMEDWGSKDIIKMNPFTVSKMPNSVANLPLRFa	(11)
		GnIH-RP-2^a^	GSSQSLLNLPQRFa	(11)
	Zebra finch	GnIH	SIKPFSNLPLRFa	(12)
		GnIH-RP-1^a^	SLNFEEMEDWRSKDIIKMNPFAASKMPNSVANLPLRFa	(12)
		GnIH-RP-2^a^	SPLVKGSSQSLLNLPQRFa	(12)

Reptiles	Anole lizard	GnIH^a^	SIKPAANLPLRFa	ENSACAG00000013069
		GnIH-RP-1^a^	SMDLESMNDWELNKIIRRTTPEMKKMAHAAVNLPLRFa	ENSACAG00000013069
		GnIH-RP-2^a^	APDVQSLSRSLANLPQRFa	ENSACAG00000013069
	Red-eared slider turtle	GnIH	SIKPVANLPLRFa	15
		GnIH-RP-1	STPTVNKMPNSLANLPLRFa	15
		GnIH-RP-2	SSIQSLANLPQRFa	15
	Chinese softshell turtle	GnIH^a^	IIKPVANLPLRFa	ENSPSIG00000017952
		GnIH-RP-1^a^	SLNFEELKDWGSKNIIKMSTPTVNKMPNSVANLPLRFa	ENSPSIG00000017952
		GnIH-RP-2^a^	TPFVKTSSQLFPNLPQRFa	ENSPSIG00000017952

Amphibians	Bullfrog	fGRP/R-RFa	SLKPAANLPLRFa	(16, 26)
		fGRP-RP-1	SIPNLPQRFa	(17)
		fGRP-RP-2	YLSGKTKVQSMANLPQRFa	(17)
		fGRP-RP-3	AQYTNHFVHSLDTLPLRFa	(17)
	Red-bellied newt	nLPXRFa-1	SVPNLPQRFa	(18)
		nLPXRFa-2	MPHASANLPLRFa	(18)
		nLPXRFa-3	SIQPLANLPQRFa	(18)
		nLPXRFa-4	APSAGQFIQTLANLPQRFa	(18)

Teleost fish	Goldfish	gfLPXRFa-1^a^	PTHLHANLPLRFa	(19)
		gfLPXRFa-2^a^	AKSNINLPQRFa	(19)
		gfLPXRFa-3	SGTGLSATLPQRFa	(19)
	Medaka	mdLPXRFa-1^a^	PLHMHANMPLRFa	XM_004073848
		mdLPXRFa-2^a^	VSNSSPNMPQRFa	XM_004073848
		mdLPXRFa-3^a^	EAPSPVLPQRFa	XM_004073848
	Grass puffer	LPXRFa-1^a^	SLDMERINIQVSPTSGKVSLPTIVRLYPPTLQPHHQHVNMPMRFa	(79)
		LPXRFa-2^a^	DGVQGGDHVPNLNPNMPQRFa	(79)
		RYa^a^	SWKVIRLCEDCSKVQGVLKHQVRYa	(79)
	Tiger puffer	LPXRFa-1^a^	SLDMERINIQVSPTSGKVSLPTIVRLYPPTLQPRHQHVNMPMRFa	(79)
		LPXRFa-2^a^	DGVQGGDHVPNLNPKMPQRFa	(79)
		RYa^a^	SWKVIRLCEDCSKVQGVLKHQVRYa	(79)

Agnathans	Sea lamprey	lLPXRFa-1a	SGVGQGRSSKTLFQPQRFa	(20)
		lLPXRFa-1b	AALRSGVGQGRSSKTLFQPQRFa	(20)
		lLPXRFa-2	SEPFWHRTRPQRFa	(20)

Protochordates	Amphioxus	PQRFa-1	WDEAWRPQRFa	(21)
		PQRFa-2	GDHTKDGWRPQRFa	(21)
		PQRFa-3	GRDQGWRPQRFa	(21)

*Ensembl or Genbank accession numbers are cited for some reptile GnIHs or medaka LPXRFa peptides. C-terminal LPXRFa (X = L or Q) sequences are underlined*.

*^a^Putative peptides hypothesized from mRNA and deduced amino-acid sequences*.

## GnIH Receptor

Yin et al. characterized the binding activity of quail GnIH and GnIH-RPs to a G-protein-coupled receptor (GPCR) GPR147. The membrane fraction of COS-7 cells transfected with quail GPR147 cDNA specifically bound GnIH and GnIH-RPs that have a C-terminal LPXRFa motif with similar affinities (22). Hinuma et al. identified a specific receptor for GnIH (RFRP) in mammals, which was identical to GPR147 and named it OT7T022 (28). In the same year, Bonini et al. reported two GPCRs for neuropeptide FF (NPFF), a neuropeptide that has a PQRFamide (PQRFa) motif at its C-terminal that modulates pain, and designated as NPFF1 (identical to GPR147) and NPFF2 (identical to GPR74) (29). LPXRFa peptide precursor gene and PQRFa peptide precursor gene are thought to have diverged from a common ancestral gene through gene duplication (20, 21). GPR147 and GPR74 genes are also paralogous (30). The binding affinities of RFRPs to GPR147 and GPR74 and their signal transduction pathways show their higher affinity to GPR147 than NPFF that has a potent agonistic activity on GPR74 (10, 29, 31), suggesting that GPR147 (NPFF1, OT7T022) is the primary receptor for GnIH (3). However, this may not apply to teleost fishes as they generally have several subtypes of GPR147 and/or GPR74 (32).

## Intracellular Signaling of GnIH Receptor

Gonadotropin-inhibitory hormone peptides suppress the production of cAMP by binding to GPR147 on the cells, suggesting that GPR147 couples to G_αi_ protein that inhibits adenylate cyclase (AC) (28, 33). Son et al. investigated the precise mechanism of GnIH cell-signaling pathway in a mouse gonadotrope cell line, LβT2 (34). Mouse RFRPs (mRFRPs) suppress GnRH-induced cAMP signaling. mRFRPs also inhibit GnRH-stimulated extracellular signal-regulated kinase (ERK) phosphorylation and gonadotropin subunit gene transcription by inhibiting the protein kinase A (PKA) pathway. Therefore, mRFRPs function as GnIH to inhibit GnRH-induced gonadotropin subunit gene transcription by inhibiting AC/cAMP/PKA-dependent ERK activation in gonadotropes (34) (Table 2).

**Table 2:** Effect of GnIH on the HPG axis of mammals.

*In vivo* (animal) or *in vitro* (pituitary or cell line)	Concentration or dose of peptides	Rout of administration, culture medium	Administration time, sample collection, measurement	Effect	Reference
***In vivo***					

Postmenopausal women	50-µg/kg/h human RFRP-3	iv	Continuous administration for 3 h	LH secretion was decreased during RFRP-3 administration	George et al. (35)

Estrous ewes	1-mg/h human RFRP-3	iv	2-h infusion	LH secretion was decreased during and after RFRP-3 administration	Clarke et al. (36)

Ovariectomized ewes treated with EB to induce LH surge	1-mg bolus + 0.5 mg/h human RFRP-3	iv	8-h infusion	EB-induced LH surge was blocked by RFRP-3	Clarke et al. (36)

Hypothalamo-pituitary disconnected ovariectomized ewes	50, 100, 200 ng GnRH during 400-µg/h human RFRP-3	iv	Blood was collected −5, 5, 10, 15, 20, 30 min after GnRH administration	RFRP-3 decreased 100-ng GnRH-induced LH secretion	Smith et al. (37)

Castrated male calves	90-µg bovine RFRP-3	iv	6 injections at 10-min intervals	LH pulse frequency was decreased during 1-h injection period	Kadokawa et al. (38)

Male rats	10, 100, 500 ng rat RFRP-3	icv	Blood was collected 20 min after administration	LH concentration was decreased by administration of 10-, 100-, or 500-ng RFRP-3	Johnson et al. (39)

Male rats	0.1, 0.5, 1, 5 nmol rat RFRP-3	icv	Blood was collected 15–120 min after administration	Total LH secretion until 120 min after administration was decreased by 5-nmol RFRP-3. FSH concentration was decreased at 15 min by 5-nmol RFRP-3. Total FSH secretion until 120 min after administration was decreased by 5-nmol RFRP-3	Pineda et al. (40)

Gonadectomized male rats	0.1, 0.5, 1, 5 nmol rat RFRP-3	icv	Blood was collected 15–120 min after administration	LH concentration was decreased at 15 min by 5-nmol RFRP-3. Total LH secretion until 120 min after administration was decreased by 1- and 5-nmol RFRP-3. Total FSH secretion until 120 min after administration was decreased by 5-nmol RFRP-3	Pineda et al. (40)

Gonadectomized male rats	10-nmol rat RFRP-3	iv	Blood was collected 15–120 min after administration	LH concentration was decreased at 60 min. Total LH secretion until 75 min after administration was decreased. FSH concentration was decreased at 60 and 75 min after administration	Pineda et al. (40)

Ovariectomized rats	1, 5 nmol rat RFRP-3	icv	Blood was collected 15–120 min after administration	LH concentration was decreased at 15 min by 1-nmol RFRP-3. Total LH secretion until 120 min after administration was decreased by 5-nmol RFRP-3	Pineda et al. (40)

Ovariectomized rats	1-µg rat RFRP-3	iv	Blood was collected 30, 60, 120 min after administration	LH concentration was decreased 120 min after administration	Murakami et al. (41)

Ovariectomized rats with E2 + P4 to induce LH surge	2.5, 25 ng/h rat RFRP-3	icv using osmotic pump	Brains were collected 2 days later at the surge peak	25-ng/h 25-ng/h RFRP-3-reduced c-Fos expression in GnRH neurons and anteroventral periventricular region that provides stimulatory input to GnRH neurons	Anderson et al. (42)

Prepubertal female mice	100, 500, 1,000 ng RFRP-3	icv	Hypothalamus and blood was collected 4 h after administration	GnRH mRNA, Kiss1 mRNA, and LH concentration was decreased by 500- and 1,000-ng RFRP-3	Xiang et al. (43)

Ovariectomized or E2-treated ovariectomized prepubertal or adult female mice	20-nmol RFRP-3	icv	Blood was collected 4 h after administration	RFRP-3 decreased LH concentration in only E2-treated ovariectomized prepubertal female mice but both E2-treated or not treated ovariectomized adult female mice	Xiang et al. (43)

Male Syrian hamsters	150, 500, 1,500, 5,000-ng Syrian hamster RFRP-3	icv	Blood was collected 30 and 120 min after administration	LH concentration was increased 30 min after administration of 500-, 15,00-ng RFRR-3. FSH concentration was increased 30 min after administration of 1,500-ng RFRR-3. Testosterone concentration was increased 120 min after administration of 1,500-ng RFRR-3	Ancel et al. (44)

Male Syrian hamsters acclimatized to SD	12-µg/day Syrian hamster RFRP-3	icv using osmotic pump	Blood was collected after 5 weeks of continuous administration	Testosterone concentration and paired testicular weight were increased to LD levels	Ancel et al. (44)

Ovariectomized Syrian hamsters	100, 300, 500 ng GnIH (icv), 600-ng GnIH (ip)	icv, ip	Blood was collected 5 (icv), 30 (icv and ip) min after administration	LH concentration was decreased 5 and 30 min after icv administration of 500-ng GnIH, and 30 min after ip administration of 600-ng GnIH.	Kriegsfeld et al. (45)

Male Siberian hamsters acclimatized to LD or SD	100- and 500-pmol Siberian hamster RFRP-1 or RFRP-3	icv	Blood was collected 5 and 30 min after administration	LH concentration was decreased 5 and 30 min after administration of 500-pmol RFRP-1, 100- and 500-pmol RFRP-3, 30 min after administration of 100-pmol RFRP-1 in LD. LH concentration was increased 30 min after administration of 500-pmol RFRP-1 or 500-pmol RFRP-3 in SD	Ubuka et al. (7)

***In vitro***					

Hypothalamic tissue of male mice	10^−7^, 10^−6^ M RFRP-3 with 10^−6^ M kisspeptin	Medium 199	After 1-h incubation medium was collected.	10^−6^ M RFRP-3 suppressed 10^−6^ M kisspeptin-induced GnRH release	Son et al. (46)

Hypothalamic tissue of female mice	10^−6^ M RFRP-3 with 10^−6^ M VIP	Medium 199	After 1-h incubation medium was collected.	10^−6^ M RFRP-3 suppressed 10^−6^ M VIP-induced GnRH release	Son et al. (46)

GFP labeled GnRH neurons of transgenic mice	0.01–1-µM GnIH or RFRP-3	aCSF	15-s application	GnIH and RFRP-3 produced a non-desensitizing hyperpolarization [IC_50_: 34 nM (GnIH), 37 nM (RFRP-3)] *via* a direct postsynaptic Ba^2+^-sensitive K^+^ current mechanism	Wu et al. (47)

GFP labeled GnRH neurons of transgenic mice	1-µM RFRP-3	aCSF	5-min application	RFRP-3 exhibited rapid and repeatable inhibitory effects on the firing rate of 41% of GnRH neurons. RFRP-3 increased the firing rate of 12% of GnRH neurons	Ducret et al. (48)

Mouse GnRH neuronal cell line (GT1–7)	10^−10^, 10^−9^, 10^−8^, 10^−7^, 10^−6^ M RFRP-1 and -3 with 10^−6^ M VIP	DMEM	6 (CRE assay) or 1 (p38, ERK assay) h application	10^−6^ M VIP-induced CRE activity was suppressed by 10^−8^, 10^−7^, 10^−6^ M RFRP-1, 3. 10^−6^ M VIP-induced p38 and ERK phosphorylation was suppressed by 10^−7^, 10^−6^ M RFRP-3	Son et al. (46)

Mouse GnRH neuronal cell line (mHypoA-GnRH/GFP)	10-, 100-nM human RFRP-3	DMEM	1-, 2-, 4-h application	GnRH mRNA expression was decreased by 100-nM RFRP-3 at 1-, 2-, 4-h application	Gojska et al. (49)

Ewe dispersed pituitary cells	10^−14^, 10^−12^, 10^−10^, 10^−8^ M human RFRP-3 with 10^−9^ M GnRH	DMEM	Medium was collected after 2-h incubation	GnRH-induced LH release was decreased by 10^−12^, 10^−10^, 10^−8^ M RFRP-3. GnRH-induced FSH release was decreased by 10^−10^, 10^−8^ M RFRP-3	Clarke et al. (50)

Gonadectomized ewe and ram dispersed pituitary cells	10^−12^, 10^−9^ M human RFRP-3 with 10^−9^ M GnRH	DMEM with 10% fetal calf serum	Medium was collected 8, 16, 24 h during incubation and finally pituitary cells were collected	GnRH-induced LH release was decreased by 10^−12^, 10^−9^ M RFRP-3 at 8-, 16-, 24-h in ewe pituitary cells. GnRH-induced LH release was decreased by 10^−12^, 10^−9^ M RFRP-3 at 8-, 16-h in ram pituitary cells. GnRH-induced FSH release was decreased by 10^−12^, 10^−9^ M RFRP-3 at 16-, 24-h in ewe pituitary cells. GnRH-induced FSH release was decreased by 10^−12^, 10^−9^ M RFRP-3 at 8-, 16-h in ram pituitary cells. GnRH-induced LHβ, FSHβ expression, ERK phosphorylation were decreased by 10^−12^, 10^−9^ M RFRP-3 in ewe and ram pituitary cells	Sari et al. (51)

Cattle dispersed pituitary cells	10^−12^, 10^−10^, 10^−8^, 10^−6^ M bovine RFRP-3 with 10^−9^ M GnRH	DMEM	Medium was collected after 2-h incubation	10^−10^, 10^−8^, 10^−6^ M RFRP-3 decreased GnRH-induced LH release	Kadokawa et al. (38)

Gonadectomized male rat pituitaries	10^−10^, 10^−8^, 10^−6^ M rat RFRP-3 with or without 10^−9^ M GnRH	DMEM	After 2-h incubation medium was collected	Basal LH concentration was decreased by 10^−8^, 10^−6^ M RFRP-3. LH concentration stimulated by GnRH was decreased by 10^−10^, 10^−8^ M RFRP-3.	Pineda et al. (40)

Female rat dispersed pituitary cells	10^−16^, 10^−14^, 10^−12^ M rat RFRP-3 with 10^−9^ M GnRH	DMEM with 10% fetal bovine serum	After 24-h incubation medium was collected	LH concentration stimulated by GnRH was decreased by 10^−12^ M RFRP-3	Murakami et al. (41)

Mouse gonadotrope cell line (LβT2)	10^−7^, 10^−6^ M RFRP-3 with 10^−7^ M GnRH	DMEM	1 h (gonadotropin subunit gene expression), 2 h (LH release) application	10^−7^ M GnRH-induced gonadotropin subunit gene expression was suppressed by 10^−6^ M RFRP-1, 3. 10^−8^ M GnRH-induced LH release was suppressed by 10^−7^, 10^−6^ M RFRP-1, 3	Son et al. (34)

Mouse gonadotrope cell line (LβT2)	10^−9^, 10^−8^, 10^−7^, 10^−6^ M RFRP-3 with 10^−7^ M GnRH	DMEM	75-min (cAMP assay), 6-h (CRE assay) or 15-min (ERK assay) application	10^−7^ M GnRH-induced cAMP production was suppressed by 10^−7^, 10^−6^ M RFRP-1, 3. 10^−7^ M GnRH-induced CRE activity was suppressed by 10^−8^, 10^−7^, 10^−6^ M RFRP-1, 3. 10^−7^ M GnRH-induced ERK phosphorylation was suppressed by 10^−6^ M RFRP-1, 3	Son et al. (34)

*aCSF, artificial cerebrospinal fluid; CRE, cAMP response element; DMEM, Dulbecco’s modified Eagle’s medium; E2, 17β-estradiol; EB, estradiol benzoate; ERK, extracellular signal-regulated kinase; FSH, follicle-stimulating hormone; GFP, green fluorescent protein; icv, intracerebroventricular administration; ip, intraperitoneal administration; iv, intravenous administration; LD, long day; LH, luteinizing hormone; P4, progesterone; SD, short day; VIP, vasoactive intestinal polypeptide*.

*Stimulatory effects on the HPG axis are underlined*.

Son et al. further investigated the signal transduction pathway that conveys the inhibitory action of GnIH in GnRH neurons by using a mouse GnRH neuronal cell line, GT1–7 (46). Although GnIH significantly suppressed the stimulatory effect of kisspeptin on GnRH release in hypothalamic culture, GnIH had no inhibitory effect on the protein kinase C (PKC) pathway stimulated by kisspeptin in GnRH neurons. On the other hand, GnIH eliminated the stimulatory effect of vasoactive intestinal polypeptide (VIP) on AC activity, p38 and ERK phosphorylation, and c-Fos mRNA expression in GT1–7. This shows the specific inhibitory mechanism of GnIH action on AC/cAMP/PKA pathway, and demonstrates a common mechanism of GnIH action in gonadotropes and GnRH neurons (34, 46) (Table 2).

## Existence of GnIH and GnIH Receptor in the HPG Axis

**Figure 1:**
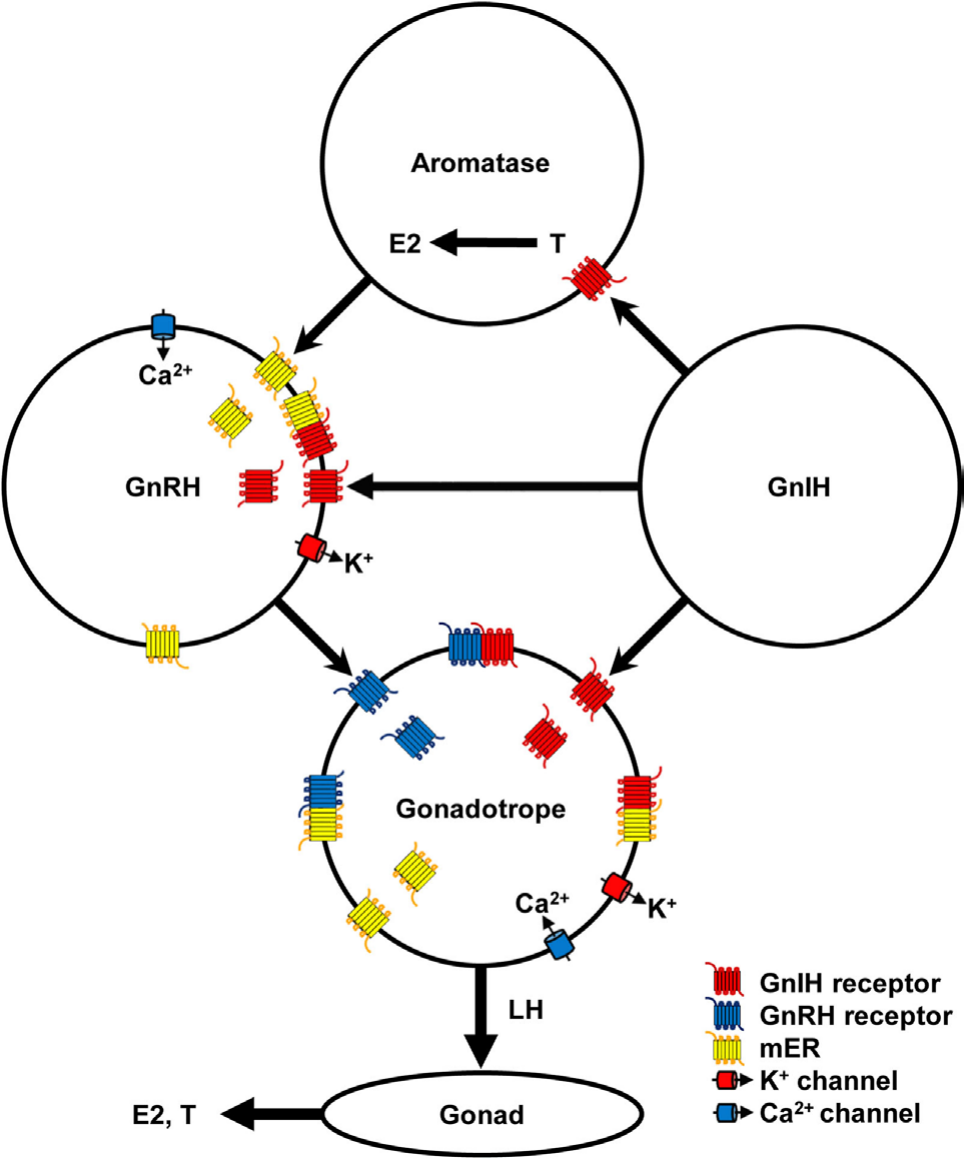
Schematic diagram of the mechanism of gonadotropin-inhibitory hormone (GnIH) action in the hypothalamic–pituitary–gonadal axis. GnIH neurons act on aromatase and gonadotropin-releasing hormone (GnRH) neurons in the hypothalamus and gonadotrope in the pituitary *via* GnIH receptor. Aromatase neurons synthesize estradiol-17β (E2) from testosterone (T) in the hypothalamus and E2 can act on GnRH neurons *via* membrane estrogen receptor (mER). GnIH stimulates K^+^ channel to hyperpolarize GnRH neurons and gonadotrope, and decrease GnRH and luteinizing hormone (LH) release, respectively. E2 stimulates Ca^2+^ channel to depolarize GnRH neurons and stimulates GnRH release. GnRH stimulates GnRH receptor and Ca^2+^ channel to depolarize gonadotrope and stimulates LH release. Low concentration of E2 inhibits Ca^2+^ channel on the gonadotrope and LH release stimulated by GnRH. LH stimulates synthesis and release of E2 and T from ovary and testis, respectively. GnIH and GnRH receptors and GPR30 (mER) belong to Class A G-protein coupled receptor family and may form heteromers to modulate ligand binding affinity and signal transduction. Binding of GnIH, GnRH, and E2 with their receptors can downregulate their cognate receptors by internalization. These complex stimulatory and inhibitory mechanisms may regulate reproductive homeostasis according to developmental and reproductive stages.

Gonadotropin-inhibitory hormone precursor mRNA is expressed in the hypothalamus of all vertebrates investigated (3). GnIH neuronal axons terminate on GnRH1 neurons in the preoptic area (POA) that terminate at the median eminence and stimulate gonadotropin secretion from the anterior pituitary gland in birds (11, 12, 52–55) (Figure 1). *In situ* hybridization of GPR147 mRNA combined with GnRH immunocytochemistry shows expression of GPR147 mRNA in GnRH1 neurons in birds (11). GnIH (RFRP) axons also terminate on the hypophysiotropic type of GnRH neurons in humans (5), monkey (6), sheep (56), hamsters (7, 45), rats (39, 57), mice (58), frog (59), zebrafish (60), and lamprey (20). Double-immunohistochemistry using GPR147 and GnRH antibodies shows GPR147 on GnRH neurons in hamsters (7) (Figure 1).

Abundant GnIH-immunoreactive (ir) fibers exist in the median eminence of humans (5), monkey (6), sheep (50), quail (1, 25, 61), sparrow (52, 62), and turtle (15). It has been clearly shown that GPR147 mRNA is expressed in the gonadotropes of human pituitary (5). GPR147-ir cells are located in the cephalic and caudal lobes of the chicken pituitary gland and they are colocalized with LHβ or FSHβ mRNA-containing cells (63). Therefore, it is likely that GnIH can directly act on the pituitary to inhibit gonadotropin synthesis and/or release from the pituitary in most birds and relatively large mammalian species (3) (Figure 1). On the other hand, GnIH may not act directly on the pituitary in some birds and rodents, as there are few or no GnIH-ir fibers in the median eminence of Rufous-winged sparrows (64), hamsters (7, 45), and rats (65). In teleost fishes, GnIH-ir fibers directly innervate the pituitary (4), which have been observed in goldfish (19), sockeye salmon (66), Indian major carp (67), sea bass (68), and tilapia (69). In the tilapia pituitary, LH cells were labeled by GnIH receptor antibody (69) (Figure 1).

## Stimulatory Effects of GnIH on the HPG Axis

An electrophysiological study has shown that RFRP-3 exhibits rapid and repeatable inhibitory effects on the firing of 41% of GnRH neurons in adult mice (48). However, stimulatory effect of RFRP-3 was observed in 12% of GnRH neurons (Table 2). No stimulatory effect of RFRP-3 on the firing of GnRH neurons was observed in diestrus mice but 18% of GnRH neurons were stimulated by RFRP-3 in proestrus female mice (48).

To understand the physiological roles of GnIH in mammalian reproduction, GnIH precursor cDNA and endogenous mature peptides have been identified in the Siberian hamster brain (7). GnIH mRNA expression and number of GnIH-ir perikarya, fibers that innervate GnRH neurons are higher in long days (LD), breeding season, compared with short days (SD), non-breeding season. Intracerebroventricular (icv) administration of hamster RFRP-1 or RFRP-3 to male Siberian hamster inhibits plasma LH concentration 5 and 30 min after administration in LD but stimulates plasma LH concentration 30 min after administration in SD (7) (Table 2). It has been also shown that central chronic administration of RFRP-3 to male Syrian hamsters adapted to SD fully restores testicular weight and plasma testosterone concentration (44, 70) (Table 2).

Moussavi et al. investigated the effect of intraperitoneal (ip) administration of goldfish LPXRFa-3 on LHβ and FSHβ subunit mRNA levels in the pituitary and serum LH concentration during gonadal cycle in goldfish (71). Circulating 17β-estradiol (E2) level is very low at early gonadal recrudescence (gr), increasing at mid-gr, very high at mid-late gr, and decreasing at late gr stages. LPXRFa-3 increased LHβ and FSHβ mRNA levels at early to mid-late and late gr, respectively. However, serum LH level is decreased by LPXRFa-3 administration at early to mid gr (Table 3). Moussavi et al. further examined the effect of ip administration of LPXRFa-3 with two native goldfish GnRHs, salmon GnRH (sGnRH) and chicken GnRH (cGnRH)-II (72). Ip administration of gfLPXRF-3 alone elevated pituitary LHβ and FSHβ mRNA levels at early and mid-gr, and only FSHβ mRNA at late gr. Coadministration of LPXRFa-3 attenuated the stimulatory effect of sGnRH on LHβ in early recrudescence, and LHβ and FSHβ mRNA levels in mid and late gr, as well as cGnRH-II-elicited increase in LHβ mRNA expression at mid and late gr. Ip administration of gfLPXRF-3 reduced serum LH levels in early and mid gr (Table 3).

**Table 3:** Effect of GnIH on the HPG axis of amphioxus, lamprey, and teleost fishes.

*In vitro* (cell line or pituitary) or *in vivo* (animal)	Concentration or dose of peptides	Culture medium, rout of administration	Administration time, sample collection, measurement	Effect	Reference
***In vivo***					

European sea bass	1, 2, 4 µg sea bass GnIH-1, 2	icv	6 h after administration brain, pituitary, and blood were collected	GnRH1 mRNA level in the brain was decreased by 1, 2, 4 µg GnIH-1. GnRH2 mRNA level in the brain was decreased by 1, 2, 4 µg GnIH-2. Kiss1 mRNA level in the brain was decreased by 2-µg GnIH-2. Kiss2 mRNA level in the brain was decreased by 2, 4 µg GnIH-2. Kiss1 receptor mRNA level in the brain was decreased by 2-µg GnIH-2. GnIH mRNA level in the brain was decreased by 1, 2 µg GnIH-2. GnIH receptor mRNA level in the brain was decreased by 1, 2 µg GnIH-2. LHβ mRNA level in the pituitary was decreased by 1, 2, 4 µg GnIH-2. FSHβ mRNA level in the pituitary was decreased by 2, 4 µg GnIH-2. GnRH receptor II1a mRNA level in the pituitary was decreased by 2, 4 µg GnIH-2. Plasma LH level was decreased by 4-µg GnIH-1 and 1-µg GnIH-2	Paullada-Salmerón et al. (73)

Goldfish	2-µg goldfish LPXRFa-3	ip	Injected twice with 12-h interval and pituitaries and blood were collected 12 h after the second injection	LHβ mRNA level was increased at early to mid-late gr. FSHβ mRNA levels was increased at early to late gr. Serum LH concentration was decreased at early to mid-gr	Moussavi et al. (71)

Goldfish	2-µg goldfish LPXRFa-3	ip	Injected twice with 12-h interval with or without 4-µg sGnRH or cGnRH-II and pituitaries and blood were collected 2 h after the second injection	LHβ level was increased by LPXRFa-3 at early to mid-gr. FSHβ mRNA levels was increased LPXRFa-3 at early to late gr. Serum LH concentration was decreased by LPXRFa-3 at early to mid-gr. LHβ mRNA level increased by sGnRH was decreased by LPXRFa-3 at early to late gr. LHβ level increased by cGnRH-II was decreased by LPXRFa-3 at mid to late gr. FSHβ mRNA level increased by sGnRH was decreased by LPXRFa-3 at mid to late gr	Moussavi et al. (72)

Sexually mature female goldfish	1-µg/g bw zebrafish LPXRFa-3	ip	Injected twice with 3-h interval and blood was collected 1 and 3 h after the second injection	Serum LH concentration was decreased by LPXRFa-3 either at 1 and 3 h after the second injection	Zhang et al. (74)

Female goldfish at late vitellogenic stage	100-ng/g bw goldfish LPXRFa-2, 3	ip	After 12-h administration hypothalamus and pituitary were collected	sGnRH mRNA level in the hypothalamus was decreased by LPXRFa-2, 3. LHβ mRNA level in the pituitary was decreased by LPXRFa-2. FSHβ mRNA level in the pituitary was decreased by LPXRFa-2, 3	Qi et al. (75)

Immature, mature male and female cinnamon clownfish	100-ng/g bw goldfish LPXRFa-3	ip	After 0, 6, 12, and 24-h administration with or without 100-ng/g bw sbGnRH brain, pituitary and blood were collected	GnIH and GnIH receptor mRNA levels in the brain were increased at 6, 12 and 24 h.^a^ GnIH and GnIH receptor mRNA levels in the brain decreased by sbGnRH were increased at 6, 12 and 24 h.^a^ sbGnRH mRNA level in the brain, plasma GnRH, FSH, LH levels, pituitary GTHα, FSHβ, LHβ mRNA levels were decreased at 6, 12 and 24 h.^a^ sbGnRH mRNA level in the brain, plasma GnRH, FSH, LH levels, pituitary GTHα, FSHβ, LHβ mRNA levels increased by sbGnRH were decreased at 6, 12 and 24 h^a^	Choi et al. (76)

Female orange-spotted grouper	100-ng/g bw grouper GnIH-I, II, III	ip	Injected twice with 6-h interval and hypothalamus and pituitary were collected 6 h after the second injection	GnRH1 mRNA level in the hypothalamus was decreased by grouper GnIH-I, II, III. GnRH3 mRNA level in the hypothalamus was increased by grouper GnIH-III. LHβ mRNA level in the pituitary was decreased by grouper GnIH-II	Wang et al. (77)

Lamprey	50, 100 µg/kg bw lamprey LPXRFa-1a, 1b, 2	ip	Injected twice with 24-h interval and brain and pituitary were collected 48 h after the second injection	Lamprey GnRH-I, III content in the brain, gonadotropin β mRNA level in the pituitary were increased by 100-µg/kg bw LPXRFa-2	Osugi et al. (20)

European sea bass	1-µg sea bass GnIH-1, 2/g bw in coconut oil	im	Injected on day 17 from October to January and blood was collected on day 22 from October to January. Brain and pituitary were collected on day 17 of February (spermiation stage)	Plasma testosterone and 11-ketotestosterone levels were decreased by sbGnIH-1, 2 in November and December (early and mid-spermatogenesis). GnRH2, sbGnIH, sbGnIH receptor, kiss1 receptor mRNA levels in the brain were increased by sbGnIH-2. LHβ mRNA level in the pituitary was decreased by sbGnIH-1 and -2. Plasma FSH level was decreased by sbGnIH-1. Plasma LH level was decreased by sbGnIH-1 and -2	Paullada-Salmerón et al. (27)

Flatfish	0.1, 1 µg/g bw flatfish GnIH-2, 3	im	Injected twice with 12-h interval and brain and pituitary were collected 4 and 8 h after the second injection	GnRH3 mRNA level in the brain was decreased by 1-µg/g bw GnIH-3 at 4 h after administration. LHβ mRNA level in the pituitary was decreased by 0.1, 1 µg/g bw GnIH-3 at 4 h after administration	Aliaga-Guerrero et al. (78)

***In vitro***					

Primary culture of male zebrafish pituitary	10^−12^, 10^−11^, 10^−10^, 10^−9^ M zebrafish LPXRFa-3	Culture media	After 18-h incubation pituitary was collected	Common α mRNA level was decreased by 10^−12^, 10^−11^, 10^−10^ M LPXRFa-3. LHβ mRNA level was decreased by 10^−11^, 10^−10^ M LPXRFa-3	Spicer et al. (60)

Primary culture of grass puffer pituitary	10^−9^, 10^−7^ M goldfish LPXRFa-1	RPMI medium	After 48-h administration pituitaries were collected	LHβ, FSHβ mRNA levels were increased by 10^−7^ M LPXRFa-1	Shahjahan et al. (79)

Primary culture of *Cichlasoma dimerus* pituitary	10^−8^, 10^−6^ M *Cichlasoma dimerus* LPQRFa-1, -2	Leibovitz L-15 medium with 10% fetal bovine serum	After 24-h incubation medium was collected	LH and FSH concentration was decreased by 10^−6^ M LPQRFa-1. FSH concentration was increased by 10^−6^ M LPQRFa-2	Di Yorio et al. (80)

Primary culture of male Nile tilapia pituitary	10^−9^, 10^−8^, 10^−7^, 10^−6^ M Pyroglutamic-tilapia LPXRFa-2	Culture medium	After 6-h incubation medium was collected	LH concentration was increased by 10^−7^, 10^−6^ M pyroglutamic-LPXRFa-2. FSH concentration was increased by 10^−6^ M pyroglutamic-LPXRFa-2	Biran et al. (81)

Dispersed goldfish pituitary cells	10^−9^, 10^−8^, 10^−7^ M goldfish LPXRFa-3	Medium 199 with 1% horse serum	After 12-h administration medium and cells were collected	LHβ mRNA level was decreased by 10^−8^ and 10^−7^ M LPXRFa-3 at early gr, increased by 10^−9^ M LPXRFa-3 at mid-gr, decreased by 10^−8^ and 10^−7^ M LPXRFa-3 at late gr. FSHβ mRNA levels was decreased by 10^−8^ and 10^−7^ M LPXRFa-3 at early gr, by 10^−9^, 10^−8^, 10^−7^ M LPXRFa-3 at mid-gr, by 10^−7^ M LPXRFa-3 at late gr. LH concentration in the media was increased by 10^−8^ M LPXRFa-3 at late gr	Moussavi et al. (71)

Dispersed female goldfish pituitary cells	10^−7^ M goldfish LPXRFa-2, 3	Medium 199 with 10% fetal bovine serum	After 12-h administration with 10^−7^ M LHRH-A cells were collected	FSHβ mRNA level increased by LHRH-A was decreased by 10^−7^ M LPXRFa-3.	Qi et al. (75)

Dispersed male sockeye salmon pituitary cells	10^−9^, 10^−7^, 10^−5^ M goldfish LPXRFa-1, 2, 3	MEM	After 2-h administration medium was collected	LH concentration in the media was increased by 10^−7^ and 10^−5^ M LPXRFa-1, 2, and 10^−9^, 10^−5^ M LPXRFa-3. FSH concentration in the media was increased by 10^−9^ and 10^−5^ M LPXRFa-1, 10^−7^, 10^−5^ M LPXRFa-2, and 10^−7^ M LPXRFa-3	Amano et al. (66)

COS-7 cells transfected with orange-spotted grouper GnIH receptor	10^−10^, 10^−9^, 10^−8^, 10^−7^, 10^−6^ M grouper GnIH-I, -II, -III	DMEM with 10% fetal bovine serum	After 24-h incubation CRE or SRC-luciferase activity was measured	Forskolin-induced CRE-luciferase activity was decreased by 10^−9^, 10^−8^, 10^−7^, 10^−6^ M grouper GnIH-I, II and 10^−6^ M grouper GnIH-III. SRE-luciferase activity was decreased by 10^−9^, 10^−7^, 10^−6^ M grouper GnIH-I	Wang et al. (77)

COS-7 cells transfected with amphioxus PQRFa receptor 1	10^−7^, 10^−6^ M amphioxus PQRFa-1, 2, 3	DMEM	After 6-h administration CRE-luciferase activity was measured	Forskolin-induced CRE-luciferase activity was decreased by 10^−6^ M PQRFa-1, 2, and 10^−7^, 10^−6^ M PQRFa-3	Osugi et al. (21)

*bw, body weight; cGnRH-II, chicken GnRH-II; CRE, cAMP response elements; DMEM, Dulbecco’s modified Eagle’s medium; FSH, follicle-stimulating hormone; gr, gonadal recrudescence; icv, intracerebroventricular administration; im, intramuscular administration; ip, intraperitoneal administration; LH, luteinizing hormone; LHRH-A, [D-Ala^6^, Pro^9^ NEt]-LHRH; MEM, minimum essential medium; sGnRH, salmon GnRH; sbGnRH, sea bream GnRH*.

*^a^Only changed at 12 and 24 h in some groups*.

*Stimulatory effects on the HPG axis are underlined*.

Ip administration of grouper GnIH-I, II, and III decreased GnRH1 mRNA level in the hypothalamus (77). However, GnRH3 mRNA level in the hypothalamus was increased by ip administration of GnIH-III. On the other hand, LHβ mRNA level in the pituitary was decreased by GnIH-II (Table 3). Ip administration of lamprey LPXRFa-2 increased GnRH-I and III content in the brain, gonadotropin β mRNA level in the pituitary [(20), Table 3]. A study in European sea bass has shown that intramuscular administration of sea bass GnIH-2 increased GnRH2 and kiss1 receptor mRNA levels in the brain (27). On the other hand, GnIH-1, 2 decreased pituitary LHβ mRNA level and plasma LH level. Plasma FSH level was only decreased by GnIH-1 (Table 3).

In addition, 48-h incubation of grass puffer pituitary with LPXRFa-1 (10^−7^ M) increased LHβ and FSHβ mRNA levels [(79), Table 3]. Although LH and FSH release from *Cichlasoma dimerus* pituitary was decreased by 24-h incubation with LPQRFa-1 (10^−6^ M), FSH release was increased by LPQRFa-2 (10^−6^ M) [(80), Table 3]. Also, 6-h incubation of Nile tilapia pituitary with pyroglutamic-LPXRFa-2 (10^−7^ and 10^−6^ M) increased LH release and pyroglutamic-LPXRFa-2 (only 10^−6^ M) increased FSH release [(81), Table 3].

Effect of goldfish LPXRFa-3 on gonadotropin synthesis and release was tested in dispersed goldfish pituitary cells collected at different gr stages (71). LHβ mRNA level was decreased by LPXRFa-3 (10^−8^ and 10^−7^ M) at early gr, but increased by LPXRFa-3 (10^−9^ M) at mid-gr, and decreased by LPXRFa-3 (10^−8^ and 10^−7^ M) at late gr. FSHβ mRNA levels was decreased by LPXRFa-3 (10^−8^ and 10^−7^ M) at early gr, by LPXRFa-3 (10^−9^, 10^−8^, 10^−7^ M) at mid-gr, and by LPXRFa-3 (10^−7^ M) at late gr. On the other hand, LH concentration in the media was increased by LPXRFa-3 (10^−8^ M) at late gr (Table 3). In dispersed pituitary cells of male sockeye salmon, LH release was increased by goldfish LPXRFa-1, 2 (10^−7^ and 10^−5^ M), and LPXRFa-3 (10^−9^ and 10^−5^ M). FSH release was increased by goldfish LPXRFa-1 (10^−9^ and 10^−5^ M), LPXRFa-2 (10^−7^, 10^−5^ M), and LPXRFa-3 (10^−7^ M) (66, Table 3).

## Possible Machnism of the Stimulatory Effects of GnIH on the HPG Axis

The mechanism of GnIH (RFRP-3) effect on the electrophysiological activity of GnRH neurons was studied in transgenic mice having vesicular glutamate transporter 2 (vGluT2)-GnRH neurons (47). GnIH and RFRP-3 produced a non-desensitizing hyperpolarization with IC_50_ values of 34 and 37 nM, respectively, in vGluT2-GnRH neurons *via* a direct postsynaptic Ba^2+^-sensitive K^+^ current mechanism (Figure 1, Table 2).

It is known that E2 secreted from the ovary negatively and positively act on the hypothalamus and pituitary to regulate the HPG axis in females. However, it is also known that E2 is synthesized from androgen by aromatase neurons in the hypothalamus (82). Recent studies have shown that E2 synthesized in the brain (neuroestrogen) directly and rapidly act on GnRH neurons *via* membrane estrogen receptor (mER) to regulate GnRH release (83, 84). GPR30 (85, 86), ERβ (87, 88) or other membrane receptors are thought to transduce the rapid effect of E2 on GnRH release (83, 89). E2 stimulates GnRH release by increasing intracellular Ca^2+^ concentration (90) and electrophysiological activity of GnRH neurons (91, 92). More recently, it has been shown that GnIH neurons terminal on aromatase neurons that express GnIH receptor and increase neuroestrogen concentration in the hypothalamus by stimulating aromatase activity in quail (93, 94). Therefore, it is possible that GnIH stimulates the electrophysiological activity of some GnRH neurones (48) by increasing neuroestrogen concentration in the hypothalamus. GnIH may further stimulate LH release that was shown in hamsters (7) by stimulating the activity of aromatase neurons and increasing neuroestrogen concentration in the hypothalamus and stimulating the electrophysiological activity of GnRH neurons and GnRH release (Figure 1).

Binding of GnRH with GnRH receptor on gonadotropes results in the activation of intracellular G_αq/11_ and phospholipases and generation of the second messengers, inositol 1-, 4-, 5-tris-phosphate, diacylglycerol, and arachidonic acid, which stimulate Ca^2+^ mobilization and PKC activity. Ca^2+^ mobilization initiates gonadotropin release (Figure 1). PKC activates mitogen-activated protein kinases (MAPKs) such as ERK, jun-N-terminal kinase, and p38 MAPK, which initiate the transcriptional activity of gonadotropin subunit genes (95). GnRH receptor also couples with G_αs_ to stimulate AC/cAMP/PKA pathway, which was shown in LβT2 cells (96) and rat gonadotropes (97). Because GnIH signaling pathway triggered by G_αi_ does not interfere with G_αq/11_ triggered pathway, GnIH may suppress gonadotropin subunit gene transcription by inhibiting AC/cAMP/PKA pathway stimulated by GnRH receptor and G_αs_ (34). GnIH may also suppress gonadotropin release by hyperpolarizing gonadotropes by activating K^+^ channel *via* GnIH receptor [(47), Figure 1].

However, recent studies of GPCR have shown that GPCR not only functions as a monomer or homodimer but also as a heterodimer with different GPCR resulting in modulation of ligand binding affinity, signal transduction, and internalization of the receptors (98, 99). It has been shown that Class A GPCRs form homo- and heteromers (100). As GnRH and GnIH receptors, and GPR30 all belong to Class A GPCR family (101), it is possible that they form heteromers in GnRH neurons and/or gonadotropes to modify the action of their ligands. Some of the stimulatory effect of GnIH on the HPG axis may be due to heteromerization of GnIH and GnRH receptor and GPR30 (Figure 1).

A recent study has shown that centrally administered GnIH can decrease plasma LH concentration in ovariectomized (OVX) prepubertal female mice that were treated with E2 but not in OVX mice that were not treated with E2 (43) (Table 2). E2 can abolish intracellular free Ca^2+^ concentration and LH release in ovine pituitary culture induced by GnRH (102). The inhibitory effect of low concentration of E2 on LH release was shown in bovine anterior pituitary mediated by GPR30 expressed on the gonadotrope (103, 104). These results suggest the modification of GnIH action by E2 in the hypothalamus and pituitary (Figure 1).

Finally, it is known for a long time that binding of GnRH with GnRH receptors is followed by aggregation, complex formation and internalization (105). Chronic administration of GnRH or antagonist administration can desensitize pituitary gonadotropes, downregulate GnRH receptor and suppress serum LH, FSH and sex-steroid levels (106–108). It is therefore possible that chronic central administration of GnIH (RFRP-3) to male Syrian hamsters adapted to SD restores testicular weight and plasma testosterone concentration by downregulation of GnIH receptor in the hypothalamus and pituitary (44, 70) (Table 2). It is also possible that stimulatory effect of GnIH on the pituitary of fish is due to downregulation of GnIH receptor by chronic administration (79, 80), high concentration of GnIH (66, 80, 81) or antagonistic effect of LPXRFa peptides of different species (66, 79) (Table 3). Inhibitory effects of GnIH on the HPG axis are shown when GnIH peptides are tested with relatively low concentrations in a shorter time frame (73–76) (Tables 2 and 3; Figure 1).

Complex mechanism may be involved in *in vivo* studies that show stimulatory and inhibitory effects of GnIH on the HPG axis in addition to downregulation of receptors and changes in the number of receptors depending on reproductive and developmental stages and endogenous sex-steroid levels (Tables 2 and 3; Figure 1). It is also important to note that GnIH peptides are produced in gonads (3, 109) and it has been shown that they have direct effects on gonadal activates in mammals (110–114), birds (115–117) and fishes (118). Most of these studies showed inhibitory effects of GnIH peptides on gonadal activities, but stimulatory activity of GnIH peptides was also shown in mouse ovary (114) and goldfish testis (118). Therefore, *in vivo* studies that showed effects of GnIH peptides on gonadal activates (Tables 2 and 3) may include direct effects of GnIH peptides on the gonads.

## Conclusion

Gonadotropin-inhibitory hormone orthologous peptides have a characteristic LPXRFamide C-terminal motif in most vertebrate species, which is critical for receptor binding. The primary receptor for GnIH is GPR147 that inhibits cAMP production in target cells. GnIH generally decreases gonadotropin synthesis and release by directly acting on the gonadotrope or by decreasing the activity of GnRH neurons. However, one study shows stimulatory effects of GnIH on the electrophysiological activity of some GnRH neurons in mice (48). Stimulatory effect of GnIH on GnRH neurons in the hypothalamus may be explained by the action of neuroestrogen synthesized in the hypothalamus by the stimulatory action of GnIH on aromatase neurons that terminate on GnRH neurons that express estrogen membrane receptor. GnIH may further stimulate LH release that was shown in hamsters by stimulating the electrophysiological activity of GnRH neurons and GnRH release (7, 44). Peripheral sex-steroid levels may also modify the action of GnIH (7, 44, 71, 72). Some of the stimulatory effects of GnIH on the HPG axis may be due to heteromerization of GnIH and GnRH receptors and GPR30 in GnRH neurons and/or gonadotropes, which modifies ligand binding and signaling transduction mechanism. Stimulatory effect of GnIH on the HPG axis may also be due to internalization of GnIH receptor by high concentration or chronic administration of GnIH or antagonistic effect of the peptides administered (20, 66, 77, 79–81). Besides pharmacological effect of administered peptides, the general inhibitory action of GnIH by decreasing cAMP concentration and inducing hyperpolarization in target cells and the additional stimulatory action of GnIH by neuroestrogen synthesis, receptor heteromerization, and internalization may have a physiological role to maintain reproductive homeostasis according to developmental and reproductive stages.

## Author Contributions

TU wrote the manuscript and IP edited the manuscript.

## Conflict of Interest Statement

The authors declare that the research was conducted in the absence of any commercial or financial relationships that could be construed as a potential conflict of interest.
